# Feasibility of mesentericoportal vein reconstruction by autologous falciform ligament during pancreaticoduodenectomy—cohort study

**DOI:** 10.1186/s12893-020-01019-9

**Published:** 2021-01-04

**Authors:** Yi Shao, Jiaojiao Feng, Yuancong Jiang, Zhenhua Hu, Jian Wu, Min Zhang, Yan Shen, Shusen Zheng

**Affiliations:** 1 Department of Hepatobiliary and Pancreatic Surgery, First Affiliated Hospital, School of Medicine, Zhejiang University, Hangzhou, 310003 China; 2 Department of Gynecologic Oncology, Women’s Hospital, School of Medicine, Zhejiang University, Hangzhou, 310006 China; 3 Key Laboratory of Organ Transplantation, Research Center for Diagnosis and Treatment of Hepatobiliary Diseases, No. 79 QingChun Road, Hangzhou, 310003 Zhejiang People’s Republic of China

**Keywords:** Pancreatic cancer, Venous reconstruction technique, Falciform ligament, Patency, Survival

## Abstract

**Background:**

Mesentericoportal vein (MPV) resection in pancreatic ductal adenocarcinoma (PDAC) surgery has become a common procedure. A few studies had described the use of falciform ligament (FL) for MPV reconstruction and received encouraging preliminary effects.

**Aims:**

This study was designed to explore the feasibility and efficacy of this technique compared with others.

**Methods:**

Patients who underwent pancreaticoduodenectomy (PD) with MPV resection for PDAC from 2009 to 2018 were enrolled. Medical records were retrospectively reviewed, MPV reconstructions using FL were distinguished and compared with other techniques.

**Results:**

146 patients underwent MPV reconstruction, and 13 received FL venoplasty. Other reconstruction techniques included primary end-to-end anastomosis (primary, n = 30), lateral venorrhaphy (LV, n = 19), polytetrafluoroethylene conduit interposition (PTFE, n = 24), iliac artery (IA) allografts interposition (n = 47), and portal vein (PV) allografts interposition (n = 13). FL group holds the advantages of shortest operation time (p = 0.023), lowest blood loss (p = 0.109), and shortest postoperative hospital stay (p = 0.125). The grouped patency rates of FL, primary, LV, PTFE, IA, and PV were 100%, 90%, 68%, 54%, 68%, and 85% respectively. Comparison displayed that FL had the highest patency rate (p = 0.008) and lowest antiplatelet/anticoagulation proportion (p = 0.000). Complications and long-term survival were similar among different techniques. The median survival time of patent group (24.0 months, 95% CI: 22.0–26.0) was much longer than that of the thrombosed (17.0 months, 95% CI: 13.7–20.3), though without significant difference (P = 0.148).

**Conclusions:**

PD with MPV resection and reconstruction by FL is safe, feasible, and efficacious, it might provide a potential benefit for patients.

## Background

With the developments in preoperative imaging, more effective neoadjuvant therapies, surgical techniques, and perioperative care, patients that were previously deemed to have borderline or even unresectable pancreatic cancer are now gaining the possibility of curative resection [1–4]. Pancreaticoduodenectomy (PD) with mesentericoportal vein (MPV) resection for the treatment of pancreatic head cancer infiltrating the MPV has now become routine in our center [5]. Despite being more and more common, MPV resection in pancreatic cancer surgery is non-standardized. And the ideal reconstruction strategy remains unclear, although various techniques have been reported. A few preliminary studies had described the application of autologous falciform ligament (FL) as a substitute for MPV reconstruction and received encouraging short-term effects [6, 7]. However in the literature, there are few results involving long-term evaluation of FL autografts as well as the advantages and disadvantages compared with other venous reconstruction techniques. In this study, we aimed to clarify the incidence of thrombosis after PD with MPV reconstruction and define the predictors as well. The operative details, antiplatelet/anticoagulation therapies, postoperative complications, and long-term survivals were also compared between FL group and other techniques.

## Methods

### Patients

From June 2009 to November 2018, patients who underwent PD with MPV resection for pancreatic ductal adenocarcinoma (PDAC) with MPV infiltration were reviewed from a prospectively maintained database. Patients with metastases detected pre- or intraoperatively, with cancer history, with celiac artery/superior mesenteric artery involvement, or without MPV infiltration were excluded from this study. All the MPV reconstructions were performed by experienced surgeons who had the qualification of liver transplantation. This study has been approved by the Research Ethics Committee of the First Affiliated Hospital, School of Medicine, Zhejiang University, Hangzhou, China and has been performed in accordance with the ethical standards as laid down in the 1964 Declaration of Helsinki and its later amendments.

### Surgical modality

Vascular invasion was evaluated by preoperative computed tomography angiography (CTA), and vascular reconstruction was planned by 3-dimensional volume-rendered images. Indication for each of the types of reconstruction was decided in a multi-disciplinary treatment meeting for each patient preoperatively. Venous reconstructions were categorized into one of 6 techniques, including (I) primary end-to-end anastomosis (primary) using a running 6-0 Prolene suture (COVIDIEN®) for closure of a short segmental resection of the vein; (II) lateral venorrhaphy (LV) where a lateral ellipse of the vein is excised and direct suture is performed; (III) FL autografts venoplasty to repair a tangential resection of the vein; (IV) Polytetrafluoroethylene vascular grafts (PTFE, GORE-TEX®) conduit interposition, (V) iliac artery (IA) allografts interposition, and (VI) portal vein (PV) allografts interposition for reconstructing a long segmental resection of the vein. PV or IA allografts were harvested from liver transplant donors and ABO-compatible was ensured in all patients. All the MPVs were reconstructed in the principle of creating a tension-free and optimal size-matched anastomosis, and systemic heparinization was not conducted. Lymphadenectomy including lymph nodes of stations 12, 13a, 13b, 17a, 17b, 5, 6, 8, 9, 14a, 14b, 16a2, and 16b1 was applied for all patients. MPV reconstructions utilizing FL autografts venoplasty were distinguished and compared with other techniques.

### Postoperative management and follow-up

Not all the patients received postoperative antiplatelet/anticoagulation therapy. The performance of antiplatelet/anticoagulation was depended on the patient’s age, general condition, platelet counts, blood coagulation functions and MHV blood flow. The protocol of subcutaneous injection of nodroparin calcium 0.4 mL per day from postoperative day 3 to 10, followed by 1 month of oral aspirin was recommended for certain cases, although the antiplatelet/anticoagulation therapy is non-standardized. MHV blood flow was observed on the third and seventh day postoperatively utilizing Doppler B-ultrasound. CTA and Doppler were reconducted 1 month, 3 months and 6 months postoperatively to justify the condition of MHV. Special personnel were responsible for regular telephone follow-up every 3-month. All subsequent treatments, relapse and survival time of the patients were investigated.

### Parameters for analysis

Patients’ data were retrospectively collected from the hospital electronic medical record. Data abstracted included demographics, operative details, and pathological parameters, especially the information of MHV reconstructions. Postoperative complications were defined according to the consensus of International Study Group of Pancreatic Surgery (ISGPS) [8]. MHV patency or occlusion was determined by postoperative imaging studies. Prognosis records were extracted from the follow-up database. All the above parameters were used for further analyzed.

### Statistical analysis

Data were analyzed by SPSS 22.0 (IBM, Armonk, NY, USA). Continuous variables were expressed as median and range, and data between two groups were compared by Student’s t-test (normal distribution and equal variance) or Wilcoxon’s test followed by Mann–Whitney U test (nonnormal distribution). Comparisons among multiple groups were performed by one-way ANOVA followed by Sidak’s post hoc multiple comparison test (normal distribution and equal variance) or Wilcoxon’s test followed by Kruskal–Wallis test (nonnormal distribution). Discrete categorical variables were presented as number and percentage and were compared by chi-square test or Fisher’s exact test, as applicable. Independent risk factors of MPV thrombosis were analyzed using logistic regression. Survival curves were analyzed using the Kaplan–Meier method and log-rank test. Independent risk factors of survival time were analyzed by Cox regression. All tests were two-tailed, a P-value < 0.05 was defined as statistical significance.

## Results

### MPV reconstructions

From June 2009 to November 2018, 702 patients underwent PD for PDAC in our center. Of these patients, 88 cases combined with metastases resection (M1), and 146 cases (out of 614 M0 cases) received MPV resections whose venous infiltrations were confirmed by postoperative pathology. 13 patients received FL venoplasty. Other reconstruction techniques included primary (n = 30), LV (n = 19), PTFE interposition (n = 24), IA allografts interposition (n = 47), and PV allografts interposition (n = 13). Patient characteristics of the cohort, including intraoperative details, pathological parameters, and postoperative characteristics, stratified by MPV reconstruction techniques, are shown in Table 1. Postoperative death within 30 days occurred in 1 (0.7%) patient from IA group who suffered a postoperative pancreatic fistula and an episode of intraabdominal bleeding requiring transfusion and relaparotomy on postoperative day 13; but still died of multi-organ failure. Mortality within 90 days, including the above case, was 2.1%.

**Table 1:** Characteristics of MPV reconstruction patients stratified by venous reconstruction techniques

Variable	FL (n = 13)	Primary (n = 30)	LV (n = 19)	PTFE (n = 24)	IA (n = 47)	PV (n = 13)	P-value
Age, year, median (range)	64 (49–74)	62 (43–79)	65 (49–80)	59 (43–80)	63 (42–82)	60 (49–71)	0.295
Male, n (%)	9 (69)	16 (53)	10 (53)	13 (54)	22 (47)	6 (46)	0.806
Operation time, min, median (range)	390 (300–610)	480 (242–813)	470 (242–720)	590 (296–921)	550 (294–835)	500 (280–704)	0.023
Blood loss, ml, median (range)	420 (200–600)	450 (150–1000)	420 (100–1000)	430 (100–1000)	550 (100–2000)	530 (200–1200)	0.109
Intraoperative transfusion, n (%)	3 (23)	5 (17)	2 (11)	1 (4)	12 (26)	2 (15)	0.295
*Postoperative pathology*							
Tumor differentiation, n (%)							0.495
Poor	10 (77)	15 (50)	12 (63)	9 (38)	27 (58)	9 (69)	
Moderate	3 (23)	14 (47)	7 (37)	15 (62)	19 (40)	4 (31)	
Well	0 (0)	1 (3)	0 (0)	0 (0)	1 (2)	0 (0)	
Tumor size, cm, median (range)	2.7 (2.0–4.0)	3.2 (1.0–6.0)	2.8 (1.0–7.0)	3.4 (1.0–7.0)	3.9 (2.0–10.0)	3.8 (2.0–8.0)	0.052
Lymph node metastasis, n (%)	9 (69)	17 (57)	7 (37)	16 (67)	31 (66)	8 (61.5)	0.305
R0 resection, n (%)	11 (85)	25 (83)	16 (84)	22 (91)	42 (89)	13 (100)	0.662
Antiplatelet/anticoagulation, n (%)	0 (0)	10 (33)	6 (32)	21 (86)	33 (70)	8 (62)	0.000
Reconstructed MPV patency	13 (100)	27 (90)	13 (68)	13 (54)	32 (68)	11 (85)	0.008
*Postoperative complications, n (%)*							
Pancreatic fistula	4 (31)	5 (17)	3 (16)	5 (21)	9 (19)	2 (15)	0.907
Bleed	2 (15)	1 (3.3)	2 (11)	2 (8)	3 (6)	1 (8)	0.811
Delayed gastric emptying	0 (0)	3 (10)	2 (11)	0 (0)	7 (15)	1 (8)	0.324
Abdominal infection	1 (8)	3 (10)	1 (5)	2 (8)	5 (11)	1 (8)	0.989
Unplanned relaparotomy	1 (8)	1 (3)	1 (5)	2 (8)	2 (4)	1 (8)	0.960
Mortality within 90 days, n (%)	0 (0)	0 (0)	0 (0)	0 (0)	2 (4)	1 (8)	0.417
Postoperative hospital stay, day, median (range)	17 (12–45)	19 (11–37)	23 (11–49)	19 (10–62)	25 (11–49)	20 (11–33)	0.125

*MPV* mesentericoportal vein, *FL* falciform ligament, *LV* lateral venorrhaphy, *PTFE* polytetrafluoroethylene, *IA* iliac artery, *PV* portal vein

### MPV patency

As shown in Table 1, comparisons among different MPV reconstruction techniques revealed that there were no significant differences in demographics and pathological parameters. The operation time of FL group was much shorter than that of other techniques (p = 0.023), moreover FL group had the lowest blood loss volume but without statistical difference (p = 0.109). R0 resection rate was similar among different techniques (p = 0.662). Antiplatelet/anticoagulation proportion of FL group was 0%, much lower than that of other techniques (p = 0.000), while MPV patency rate of FL group reached 100%, highest among all the techniques (p = 0.008). Postoperative complications, including pancreatic fistula, bleeding, delayed gastric emptying, abdominal infection, unplanned relaparotomy, and mortality within 90 days, were similar among different techniques. Whereas, FL group had the shortest postoperative hospital stay, though without significant difference compared to other techniques (p = 0.125).

In order to define the predictors of thrombosis after MPV reconstruction, data were reorganized and stratified by MPV patent or thrombosed (Table 2). There were significant differences in venous reconstruction techniques, operation time, blood loss, lymph node metastasis, pancreatic fistula, postoperative bleeding, unplanned relaparotomy, and postoperative hospital stay between the two groups (Table 2). Multiple logistic regression analysis of these factors showed that prolonged operation time was an independent risk factor for thrombosis (Table 3).

**Table 2:** Characteristics of MPV reconstruction patients stratified by venous patent or thrombosed

Variable	All patients (n = 146)	Patent (n = 109)	Thrombosed (n = 37)	P-value
Age, year, median (range)	62 (42–82)	63 (42–82)	60 (43–78)	0.052
Male, n (%)	76 (52.1)	58 (53.2)	18 (48.6)	0.631
MPV reconstruction technique, n (%)				0.008
FL	13 (8.9)	13 (11.9)	0 (0.0)	
Primary	30 (20.5)	27 (24.8)	3 (8.1)	
LV	19 (13.0)	13 (11.9)	6 (16.2)	
PTFE	24 (16.4)	13 (11.9)	11 (29.7)	
IA	47 (32.2)	32 (29.4)	15 (40.5)	
PV	13 (8.9)	11 (10.1)	2 (5.4)	
Operation time, min, median (range)	515 (242–921)	450 (242–790)	680 (250–921)	0.000
Blood loss, ml, median (range)	463 (100–2000)	415 (100–1500)	581 (100–2000)	0.037
Intraoperative transfusion, n (%)	25 (17.1)	15 (13.8)	10 (27.0)	0.064
*Postoperative pathology*				
Tumor differentiation, n (%)				0.514
Poor	82 (56.2)	63 (57.8)	19 (51.4)	
Moderate	62 (42.5)	44 (40.4)	18 (48.6)	
Well	2 (1.4)	2 (1.8)	0 (0.0)	
Tumor size, cm, median (range)	3.4 (1–10)	3.4 (1–10)	3.4 (1–8)	0.919
Lymph node metastasis, n (%)	88 (60.3)	60 (55.0)	28 (75.7)	0.027
R0 resection, n (%)	129 (88.4)	97 (89.0)	32 (86.5)	0.682
*Postoperative complications, n (%)*				
Pancreatic fistula	28 (19.2)	16 (14.7)	12 (32.4)	0.018
Bleed	11 (7.5)	5 (4.6)	6 (16.2)	0.031
Delayed gastric emptying	13 (8.9)	10 (9.2)	3 (8.1)	0.844
Postoperative abdominal infection	13 (8.9)	7 (6.4)	6 (16.2)	0.071
Unplanned relaparotomy, n (%)	8 (5.5)	3 (2.8)	5 (13.5)	0.025
Mortality within 90 days, n (%)	3 (2.1)	2 (1.8)	1 (2.7)	1.000
Antiplatelet/anticoagulation, n (%)	78 (53.4)	57 (52.3)	21 (56.8)	0.638
Postoperative hospital stay, day, median (range)	21.4 (10–62)	19.5 (10–49)	25.7 (10–62)	0.026

*MPV* mesentericoportal vein, *FL* falciform ligament, *LV* lateral venorrhaphy, *PTFE* polytetrafluoroethylene, *IA* iliac artery, *PV* portal vein

**Table 3:** Multiple logistic regression analysis of the risk factors of MPV thrombosis

Risk factors	P value	Odds ratio	95% CI
Operation time	0.000	1.110	1.005–1.215

*MPV* mesentericoportal vein, *CI* confidence interval

### Survival analysis

**Fig. 1:**
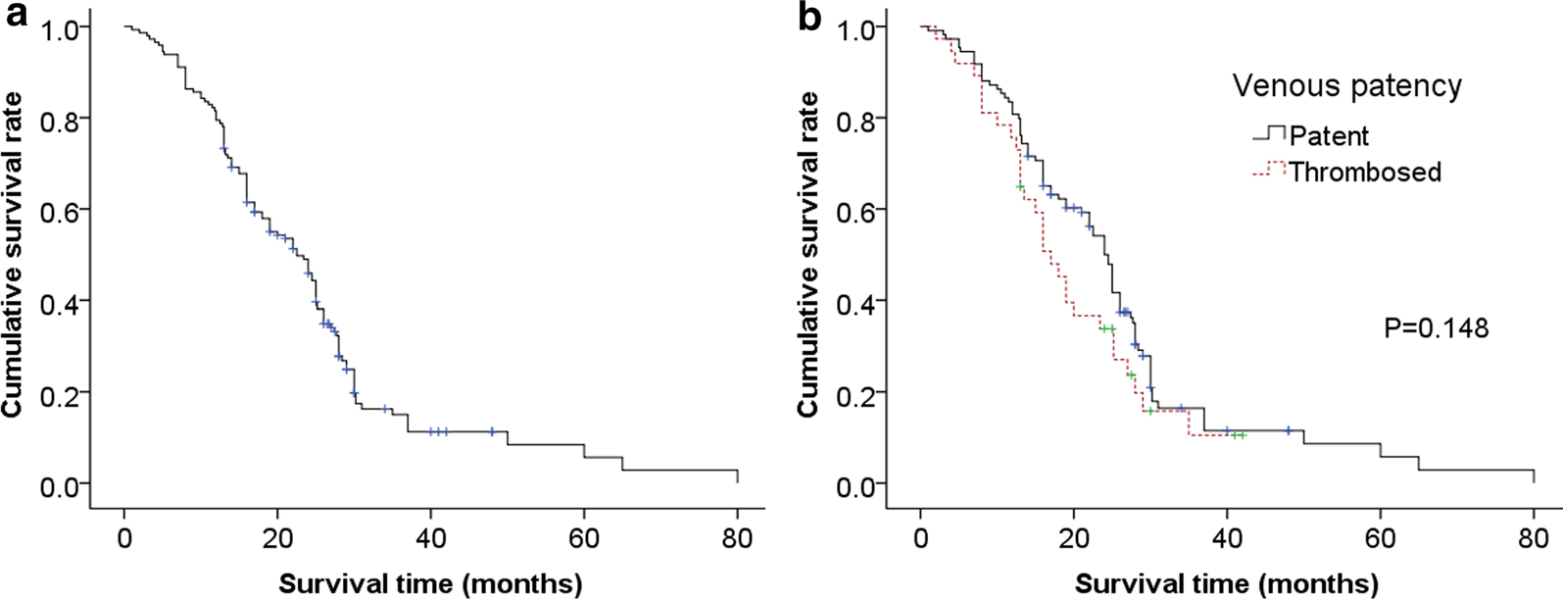
Analysis of overall survival of 146 patients (**a**) and comparison of survival curves between the patent and the thrombosed (**b**) by the Kaplan–Meier method

**Fig. 2:**
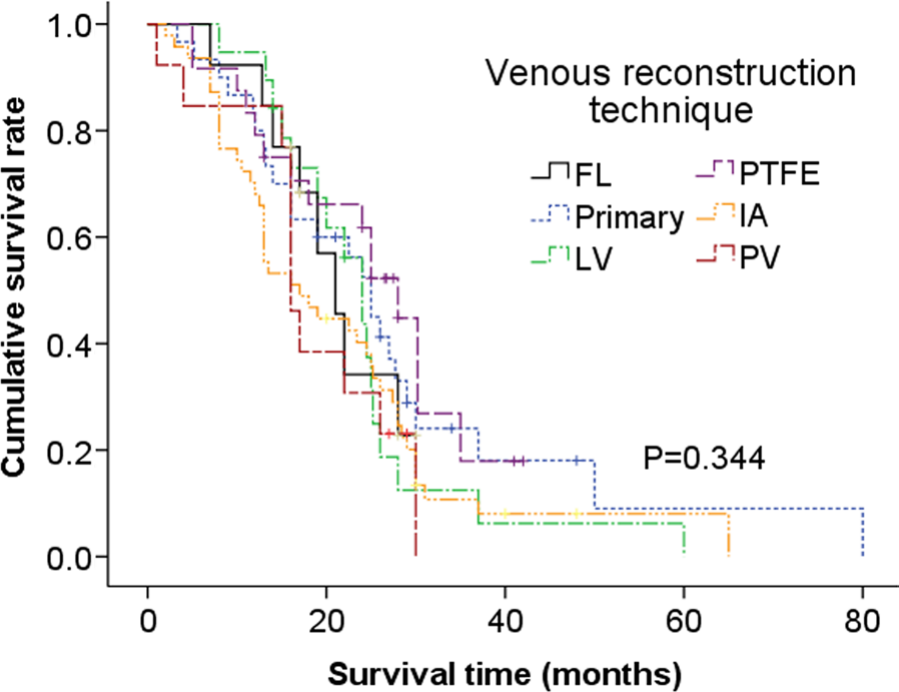
Comparison of survival curves among different venous reconstruction techniques by the Kaplan–Meier method

The overall 1-, 2- and 3-year survival rates of the series were 79.5%, 45.9% and 15.0%, respectively, with a median survival time of 22.5 months (95% CI: 18.2–26.8, Fig. 1a). A comparison of survival curves between patent and thrombosed group showed that the median survival time of patent group (24.0 months, 95% CI: 22.0–26.0) was much longer than that of the thrombosed (17.0 months, 95% CI: 13.7–20.3), although without significant difference (P = 0.148, Fig. 1b). Also, the Kaplan–Meier estimate of survival time demonstrated that the differences among each venous reconstruction techniques were not statistically significant (p = 0.344, Fig. 2).

**Fig. 3:**
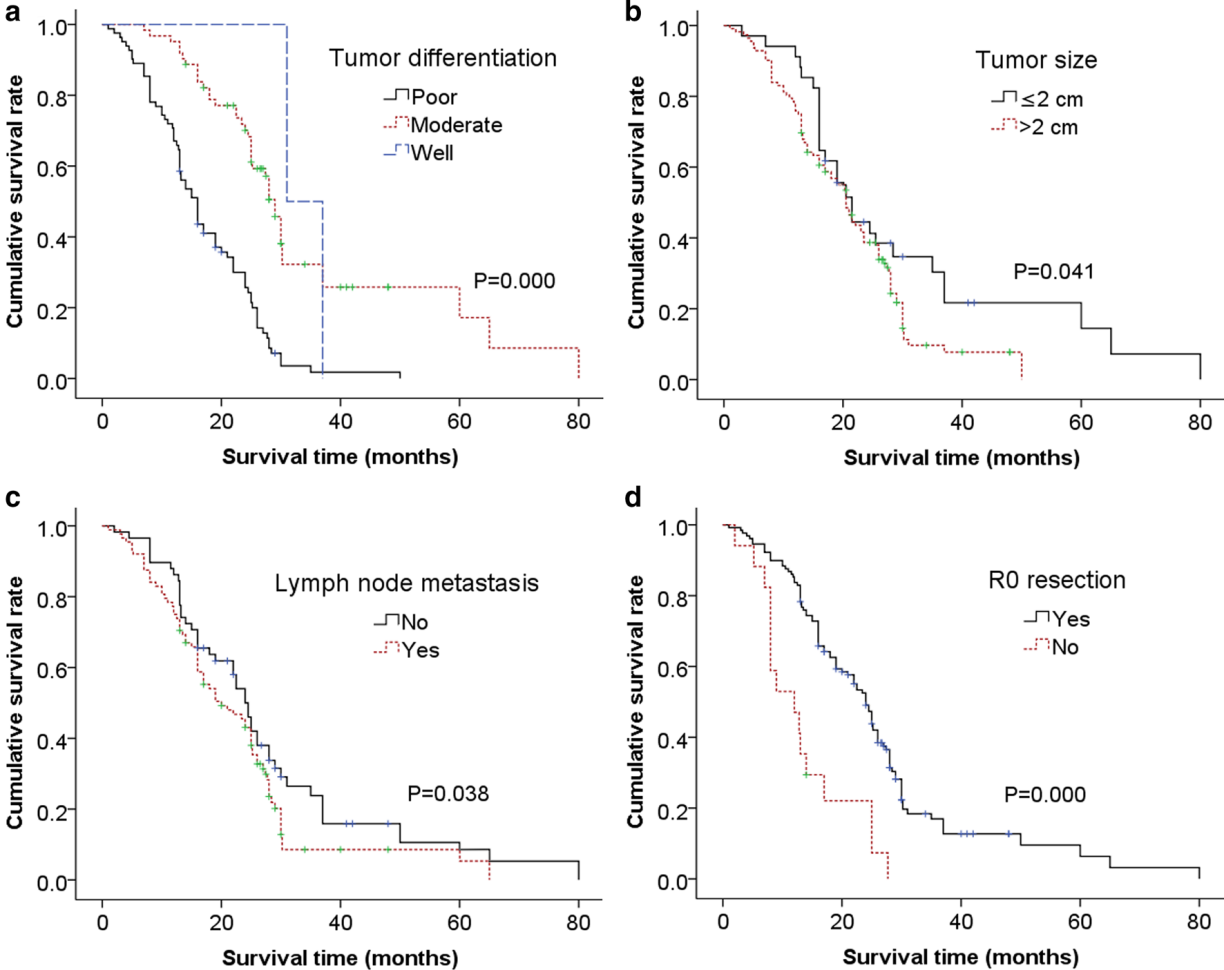
Kaplan–Meier estimate showed that poor differentiation (**a**), tumor size > 2 cm (**b**), lymph node metastasis (**c**), and non R0 resection (**d**) are risk factors for survival time

Poor differentiation, tumor size (> 2 cm), lymph node metastasis, and non R0 resection were considered to be adverse factors for survival time (Fig. 3). Cox regression analysis showed that poor differentiation and non R0 resection were independent risk factors for survival time (Table 4).

**Table 4:** Cox regression analysis of multiple factors affecting the survival time in PDAC

Risk factors	P value	Relative risk	95% CI
Poor differentiation	0.000	3.597	2.396–5.400
Non R0 resection	0.001	2.495	1.425–4.368

*PDAC* pancreatic ductal adenocarcinoma, *CI* confidence interval

## Discussion

MPV resection is becoming more and more common in PDAC surgeries. Although most of the existing data are from retrospective and heterogeneous studies, this does not prevent surgeons from treating PDAC with MPV resection as a routine procedure [1, 2, 4]. With proper patient selection, the need for MPV resection in PDCA patients does not significantly influence survival time if R0 resection can be acquired [5].

Various MPV reconstruction techniques have been described, including the use of synthetic PTFE grafts [9], bovine pericardium, allografts [5, 10], autografts [7, 11, 12], as well as different segmental or tangential resections with primary end-to-end anastomosis or venorrhaphy [13, 14], resulting in an overall patency rate between 70 and 90% [9–14]. Each technique has its own advantages and disadvantages, so far the optimal reconstruction strategy is still unclear.

Recently, a few preliminary studies had reported the use of autologous FL as a substitute for MPV reconstruction and received inspiring short-term effects [6, 7]. While the main limitations of the former studies were the small load of examined cases, the lack of comparison with other reconstruction techniques, and the impact on long-term prognosis. Thus in this study, we enrolled 146 cases of PD with MPV resection for PDAC patients, among which 13 were reconstructed by FL, 30 by primary, 19 by LV, 24 by PTFE, 47 by IA, and 13 by PV. Compared with other techniques, FL group hold the advantages of shortest operation time (p = 0.023), lowest blood loss (p = 0.109), and shortest postoperative hospital stay (p = 0.125). Moreover FLs can be rapidly harvested without additional injury, and are also easy to access, which can be temporarily used in emergencies or unplanned situations. Besides, FL is autologous, hence it has better biocompatibility and less rejection reaction than other substitutes. Therefore antiplatelet/anticoagulation was not performed in this group, still resulting in a patency rate of 100%. Further, the grouped patency rates of other techniques were calculated as well, those were 90%, 68%, 54%, 68%, and 85% for primary, LV, PTFE, IA, and PV respectively. Comparison displayed that FL had the highest patency rate (p = 0.008) and lowest antiplatelet/anticoagulation proportion (p = 0.000).

According to the reconstructed MPV patent or not, data were reorganized and divided into two groups. Analysis demonstrated that prolonged operation time was an independent risk factor for thrombosis, suggesting an increased difficulty in the surgical procedure as well. Comparison of postoperative complications revealed that the incidence rates were similar among different techniques, illustrating that FL bore equal surgical riskiness as other techniques. Besides patch use, FL autografts were also used for tubular reconstructions in two patients of pancreatic neuroendocrine tumors and obtained perfect results (these data were not included in this study). Due to the data limitation, the use of FL for tubular reconstruction can not be well judged yet [7]. In our experiences, FL autografts were most used for patch venoplasty. And primary end-to-end anastomosis was recommended for short segmental resections. Supposing the resected segment is too long to be anastomosed directly, conduit graft bridging using PV allograft can be the first choice. In the case of vascular resection involving bifurcation, venous reconstruction using “Y” shaped IA allograft should be the first choice. Thus, the FL autograft is suitable for many reconstruction situations, but not all cases. Besides, it might provide an alternative choice for surgeons.

Interestingly, we found that there was no difference in anticoagulation therapy between the patent group and the thrombosed (p = 0.638). It was seemed that anticoagulation therapy didn’t provide any preventive or protective benefit for thrombosis. Our discovery is similar to previous research results [13, 15]. However, due to the technical complexity of these operations and the heterogeneity of existing data, it is difficult to standardize practice details without randomized prospective trials [13, 15]. A limitation of this study is that specific schemes and duration of anticoagulation were not included in the analysis.

Survival analysis disclosed that the difference of survival time among each venous reconstruction techniques was not significant. Whereas the long-term survival of patent group was much better than that of the thrombosed, although without statistical difference. Multiple factors analysis exhibited that poor differentiation and non R0 resection were independent risk factors of survival. So striving for R0 resection and choosing appropriate technique to ensure the patency of reconstructed MPV are of critical significances for improving long-term survival of PDAC [16].

## Conclusions

MPV reconstruction using FL autograft is safe, feasible and efficacious, it may expand surgical indications, improve R0 resection rate compared with traditional procedures, and provide an alternative choice for surgeons.

## Data Availability

The datasets used and analysed during this study are available from the corresponding author upon reasonable request.

## Abbreviations

CI: Confidence interval
CTA: Computed tomography angiography
FL: Falciform ligament
IA: Iliac artery
LV: Lateral venorrhaphy
MPV: Mesentericoportal vein
PD: Pancreaticoduodenectomy
PDAC: Pancreatic ductal adenocarcinoma
PTFE: Polytetrafluoroethylene
PV: Portal vein
